# A case report of severe pneumonia caused by *Aeromonas dhakensis* infection complicated with severe atrial septal defect

**DOI:** 10.3389/fmed.2024.1476864

**Published:** 2024-11-18

**Authors:** Jun Sha, Jie Shao, Sheng Lu, Mengmeng Zhang, Cheng Gu, Yimai Deng, Jianfeng Zhang, Yufeng Feng

**Affiliations:** ^1^Intensive Care Unit, Changshu No. 2 People’s Hospital, Suzhou, China; ^2^Changshu Medicine Examination Institute, Suzhou, China

**Keywords:** *Aeromonas dhakensis*, severe pneumonia, atrial septal defect, mNGS, ECMO

## Abstract

*Aeromonas dhakensis* is an increasingly recognized human pathogen in recent years and was first isolated and reported in a sample of childhood diarrhea in Bangladesh. More and more cases of *Aeromonas dhakensis* infection have been reported in recent years. Here we report a case of severe pneumonia caused by *Aeromonas dhakensis* with severe atrial septal defect. The patient, a 56-year-old male, was admitted to the hospital with severe hypoxemia and severe septic shock. Detection of the patient’s bronchoalveolar lavage fluid (BALF) and peripheral blood by the metagenomic next generation sequencing (mNGS) indicated *Aeromonas dhakensis* infection.

## Introduction

*Aeromonas dhakensis* is a highly pathogenic human pathogen discovered in recent years (1). *Aeromonas dhakensis* is a Gram-negative bacillus and widely distributed in water environments (1). *Aeromonas dhakensis* is extremely virulent and can cause severe sepsis and multiple organ failure in a short time (2), with a 14-day sepsis-related mortality rate of 25.5% (3). *Aeromonas dhakensis* has been reported to have multiple virulence factors and its strains have cytotoxic activity against human blood cell lines (4). *Aeromonas dhakensis*, for example, produces a cytotoxic enterotoxin (5) and also secretes a pathogenic exotoxin A (6). Clinically, it can lead to the most common intestinal infections in patients, but also can lead to extremely serious invasive parenteral infections, such as lung infections, biliary tract infections, and soft tissue infections. Patients with compromised immunity are more susceptible to infection of *Aeromonas dhakensis*, which can lead to severe sepsis and organ failure (7). Here, we report a case of a 56-year-old man diagnosed with *Aeromonas dhakensis* pneumonia with severe atrial septal defect.

## Case report

**Figure 1:**
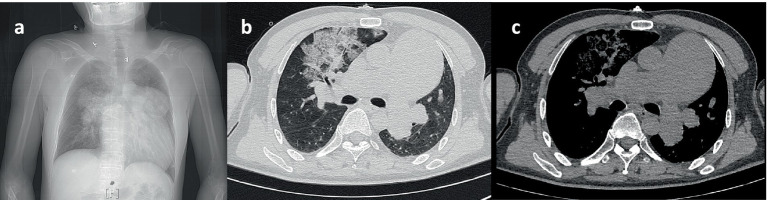
(a–c) The chest computer tomography (CT) scan displayed a double lung infection.

**Figure 2:**
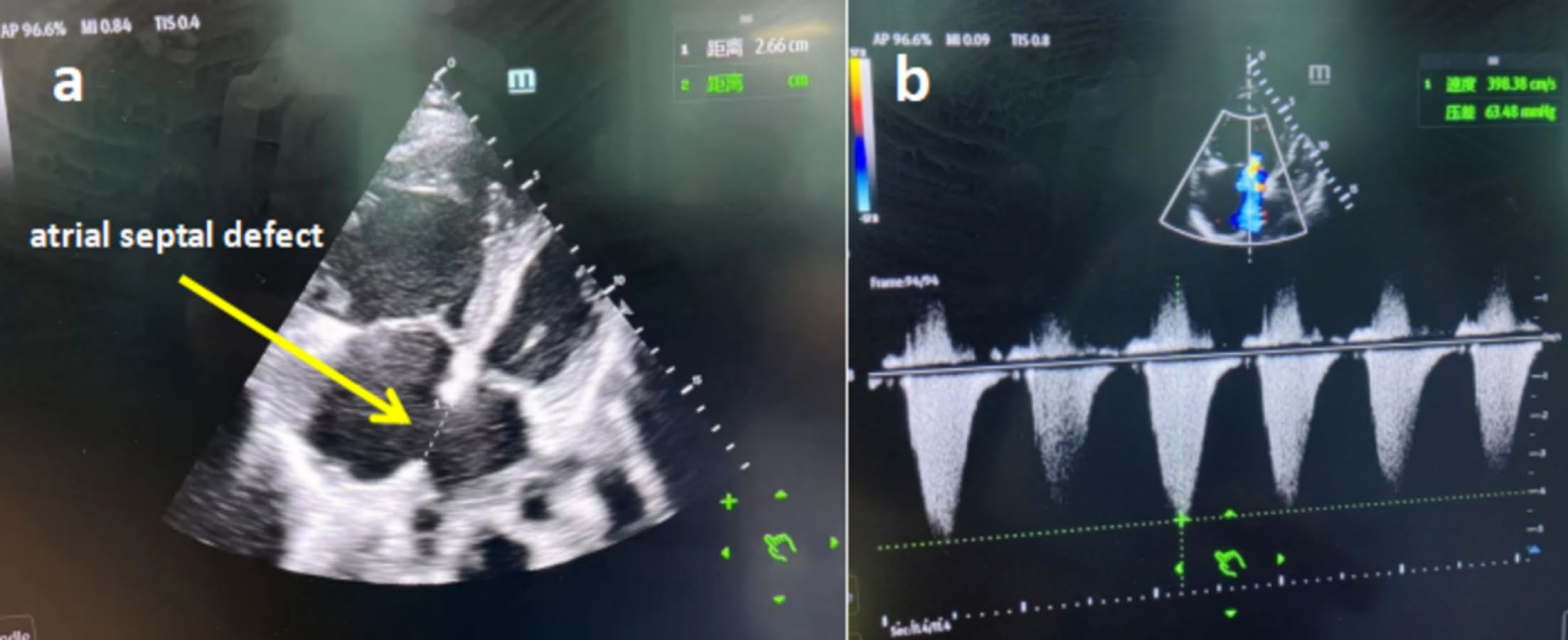
(a) Cardiac ultrasound showed the patient with severe atrial septal defect. (b) Cardiac ultrasound showed the patient with severe pulmonary hypertension.

A 56-year-old male presented to the hospital due to fever with chest tightness and asthma for 1 day. The patient was admitted to ICU after endotracheal intubation with mechanical ventilation due to severe hypoxemia. The patient had a history of atrial septal defect for more than 30 years. A blood routine examination showed that white blood cell count was 2.0 × 10^9^/L, the percentage of neutrophils was 85.8%, and platelet count was 43 × 10^9^/L. C-reactive protein was 3.8 mg/L. Blood gas analysis showed that the oxygenation index was 39.5 mmHg. Procalcitonin was 91.8 ng/mL. The chest computer tomography (CT) scan displayed inflammation in both lungs, mainly in the upper lobe of the right lung (Figure 1). Cardiac ultrasound revealed that the atrial septal defect was 2.66 cm, and the pulmonary artery pressure is about 80 mmHg (Figure 2).

The patient was diagnosed with severe pneumonia, severe acute respiratory distress syndrome (ARDS), septic shock, sepsis, severe atrial septal defect, and severe pulmonary hypertension. The patient’s empiric anti-infective therapy was omadacycline in combination with imipenem. The patient received the treatment of veno-arterio-venous extracorporeal membrane oxygenation (VAV-ECMO) immediately due to poor finger pulse oxygen and blood pressure. However, after ECMO treatment, the patient’s finger pulse oxygen was still poor. Sildenafil, ambrisentan and inhaled NO were given to reduce pulmonary arterial pressure, considering the patient had severe atrial septal defect and pulmonary hypertension, and severe pulmonary shunt. At the same time, the metagenomic next generation sequencing (mNGS) tested by the company Nanjing KingMed for clinical laboratory through Illumina MiSeq sequencing platform was used to detect the pulmonary alveolar lavage fluid (BALF) and peripheral blood to identify the infectious pathogen. Direct microscopic examination of BALF and peripheral blood showed Gram-negative bacteria.

**Figure 3:**
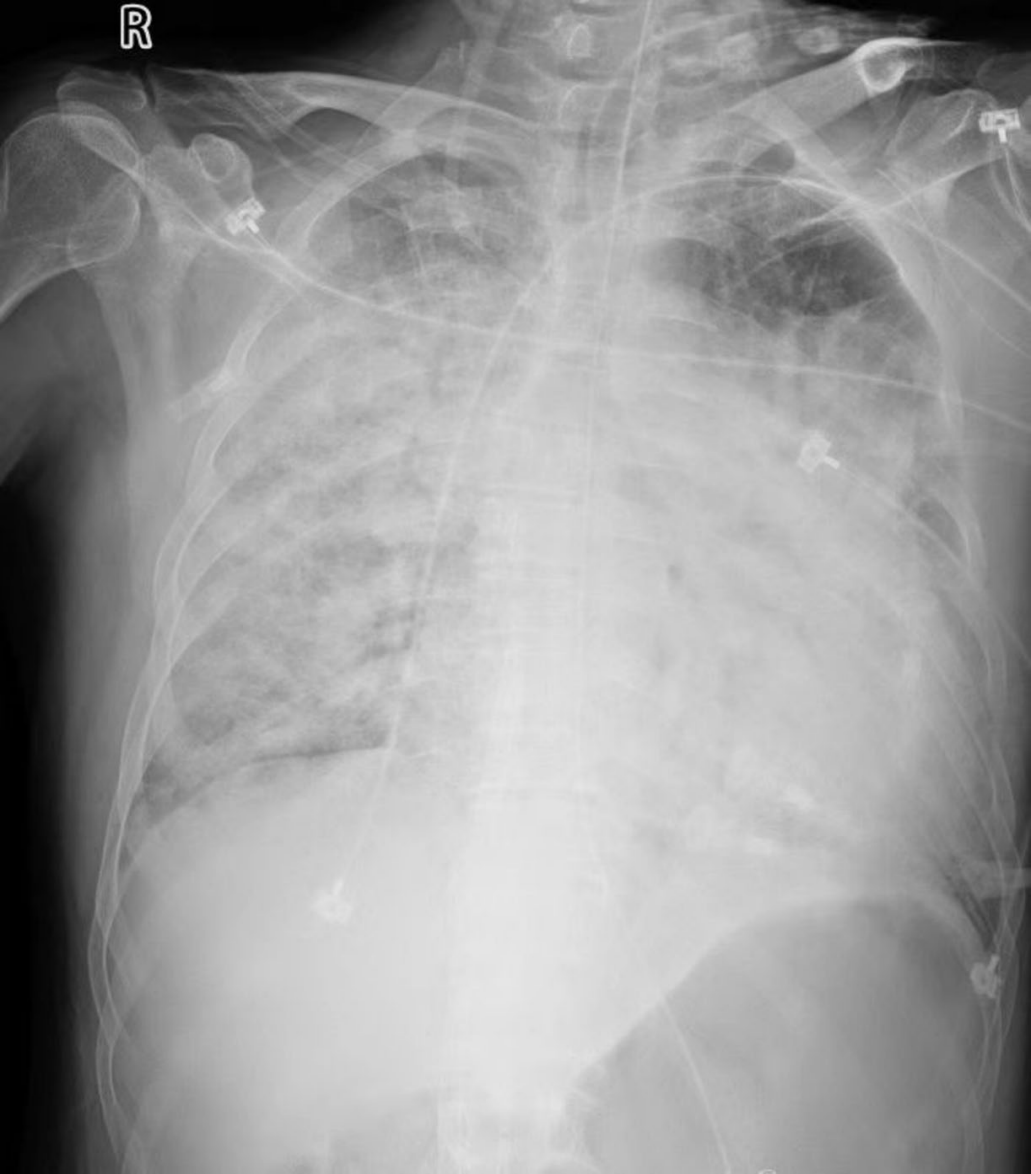
Chest X-ray of the patient after effective anti-infective treatment.

**Figure 4:**
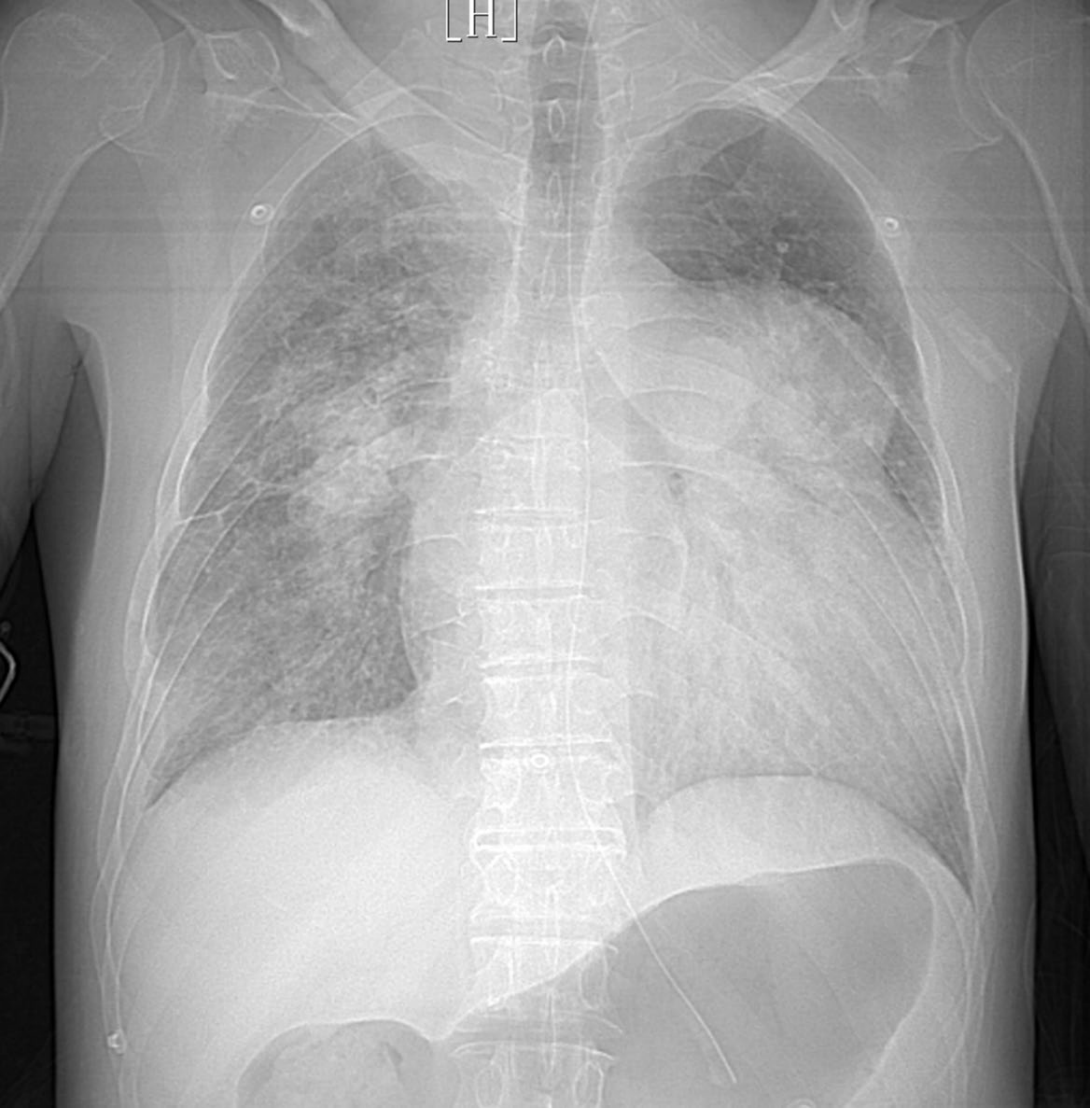
Chest X-ray of the patient after withdrawing ventilator successfully.

On the third day of hospitalization, the results of the mNGS showed that *Aeromonas dhakensis* was positive and the relative abundance of *Aeromonas dhakensis* was 95.68%. According to the results of drug sensitivity test, *Aeromonas dhakensis* was sensitive to omoxycycline and imipenem. Therefore, the anti-infection treatment plan would not be adjusted. After active treatment for 2 weeks, the inflammatory index of the patient decreased significantly, and the patient’s consciousness became clear. However, due to pulmonary interstitial changes caused by infection and severe pulmonary hypertension, the patient still presented with moderate to severe respiratory failure and was unable to leave the ventilator (Figure 3). After 2 months of mechanical ventilation and rehabilitation exercise, the patient was successfully removed from the ventilator and transferred out of ICU (Figure 4). But the pulmonary interstitial changes caused by infection could not return to normal.

## Discussion

*Aeromonas* is a Gram-negative bacterium. Human infections are usually caused by *Aeromonas hydrophila*, *Aeromonas veronii* biovar sobria, and *Aeromonas caviae* (8). *Aeromonas* is widely distributed in various kinds of freshwater waters, and the infection of *Aeromonas* in humans is usually through direct contact with water containing pathogenic bacteria (9). People with chronic underlying disease and low immunity are more susceptible to *Aeromonas*. *Aeromonas* can cause gastrointestinal tract, skin and soft tissue, respiratory infections, nervous system and biliary tract infections (10). The clinical manifestations of *Aeromonas* infection are usually rapid onset, severe symptoms and severe sepsis. It has been reported that the initial symptoms of *Aeromonas* infection may be diarrhea, cough, expectoration and hemoptysis (2).

*Aeromonas dhakensis* was previously considered a subspecies of *Aeromonas hydrophila* (11), which was first isolated from a sample of childhood diarrhea in Bangladesh (12). But according to the latest microbiology studies, whole genome sequence analyses unambiguously confirmed that *Aeromonas dhakensis* reached the level of species (13). *Aeromonas dhakensis* can also cause infections in the digestive, respiratory, urinary, hepatobiliary and skin and soft tissues (3). The mortality rate of *Aeromonas dhakensis* is much higher than other *Aeromonas* species (14), because *Aeromonas dhakensis* contains multiple pathogenic genes and can produce multiple exotoxins. *Aeromonas dhakensis* strains have toxic effects on human blood cell lines, which may result in a reduction in blood cell lines (15, 16). Up to now, the specific pathogenesis of *Aeromonas dhakensis* remains unclear (17).

*Aeromonas dhakensis* is sensitive to third or fourth generation cephalosporins, aminoglycosides, fluoroquinolones, and tetracyclines (18). *Aeromonas dhakensis* has been reported to produce a variety of β-lactamases resulting in resistance to a variety of penicillins, cephalosporins and even carbapenems (19). Clinicians should be cautious about the use of cephalosporins alone for anti-infective treatment if clinically suspected or confirmed *Aeromonas dhakensis* infection (20).

## Conclusion

Clinically, we observed that the infection of *Aeromonas dhakensis* led to the rapid onset of severe septic shock and the possible complications of multiple organ failure Including heart failure, renal failure, respiratory failure, and decreased blood cell line. *Aeromonas dhakensis* is not a common pathogen in community-acquired pneumonia, but it can cause rapid onset, severe symptoms and multiple organ dysfunction in patients. At this time, clinicians should think of the possibility of a *Aeromonas dhakensis* infection. In this case, we used ECMO early to maintain the patient’s vital signs, which bought time and opportunity for effective treatment.

## Data Availability

The original contributions presented in the study are included in the article/supplementary material, further inquiries can be directed to the corresponding author.
